# A Mathematical Model of the Dynamics of Cytokine Expression and Human Immune Cell Activation in Response to the Pathogen *Staphylococcus aureus*

**DOI:** 10.3389/fcimb.2021.711153

**Published:** 2021-11-10

**Authors:** Kian Talaei, Steven A. Garan, Barbara de Melo Quintela, Mette S. Olufsen, Joshua Cho, Julia R. Jahansooz, Puneet K. Bhullar, Elliott K. Suen, Walter J. Piszker, Nuno R. B. Martins, Matheus Avila Moreira de Paula, Rodrigo Weber dos Santos, Marcelo Lobosco

**Affiliations:** ^1^ Center for Research and Education in Aging, University of California, Berkeley, Berkeley, CA, United States; ^2^ Lawrence Berkeley National Laboratory, Berkeley, CA, United States; ^3^ Department of Integrative Biology, University of California, Berkeley, Berkeley, CA, United States; ^4^ Department of Computer Science, Federal University of Juiz de Fora, Juiz de Fora, Brazil; ^5^ Department of Mathematics, North Carolina State University, Raleigh, NC, United States; ^6^ College of Chemistry, University of California, Berkeley, Berkeley, CA, United States; ^7^ Mayo Clinic Alix School of Medicine, Scottsdale, AZ, United States; ^8^ Department of Molecular and Cell Biology, University of California, Berkeley, Berkeley, CA, United States

**Keywords:** cytokines, mathematical modeling, immune response, immune system, *Staphycoccus aureus*, cytokine response, cell activation

## Abstract

Cell-based mathematical models have previously been developed to simulate the immune system in response to pathogens. Mathematical modeling papers which study the human immune response to pathogens have predicted concentrations of a variety of cells, including activated and resting macrophages, plasma cells, and antibodies. This study aims to create a comprehensive mathematical model that can predict cytokine levels in response to a gram-positive bacterium, *S. aureus* by coupling previous models. To accomplish this, the cytokines Tumor Necrosis Factor Alpha (TNF-*α*), Interleukin 6 (IL-6), Interleukin 8 (IL-8), and Interleukin 10 (IL-10) are included to quantify the relationship between cytokine release from macrophages and the concentration of the pathogen, *S. aureus, ex vivo*. Partial differential equations (PDEs) are used to model cellular response and ordinary differential equations (ODEs) are used to model cytokine response, and interactions between both components produce a more robust and more complete systems-level understanding of immune activation. In the coupled cellular and cytokine model outlined in this paper, a low concentration of *S. aureus* is used to stimulate the measured cellular response and cytokine expression. Results show that our cellular activation and cytokine expression model characterizing septic conditions can predict *ex vivo* mechanisms in response to gram-negative and gram-positive bacteria. Our simulations provide new insights into how the human immune system responds to infections from different pathogens. Novel applications of these insights help in the development of more powerful tools and protocols in infection biology.

## 1 Introduction

The human immune system consists of the innate and adaptive immune response. Innate immunity comprises multiple lines of defense beginning with skin, saliva, and various secretions, and ending with non-specific leukocytes, while adaptive immunity refers to a long-term specific response initiated to eliminate a specific pathogen (Kim et al., 2007; Mirzaei et al., 2020). The immune system leukocytes are usually in the form of neutrophils, macrophages, eosinophils, or natural killer cells. Their primary function is to perform phagocytosis of pathogens and cell debris through engulfment and chemical degradation (Badwey and Karnovsky, 1980). If the innate immune system is insufficient in eliminating the pathogen, it activates the adaptive immune system, which is primarily composed of B cells and T cells, known for their specificity in function (Zhang and An, 2007). B cells produce and recruit antibodies that tag antigens on infected cells for T cells to subsequently destroy (Winer et al., 2011). In addition, both B and T cells play a role in the production of cytokines, which are small signaling proteins released by leukocytes that facilitate communication between immune cells.

One such signaling protein is TNF-α, which is instrumental to the acute phase reaction during an inflammatory response. In response to sepsis, TNF-α, a regulator of immune cells, functions by upregulating other cytokines, including IL-1 and IL-6 (Feuerstein et al., 1994). IL-6 is involved in inflammation and homeostatic processes, but though it is primarily a proinflammatory cytokine, it can also act as an anti-inflammatory cytokine through its inhibitory effects on TNF-α. During inflammation and the delay in apoptosis of T cells, IL-6 is critical for the recruitment of T cells and the production of B and T cells (Fournier and Philpott, 2005). Similarly, IL-8 is also involved in the recruitment of T cells, as well as basophils and neutrophils (Holmes et al., 1991). IL-8 has been shown to be induced by TNF-α and inhibited by IL-10 (Yao et al., 1996; Osawa et al., 2002) which is an anti-inflammatory cytokine critical in the regulation of immune responses (de Waal Malefyt et al., 1991). In particular, IL-10 limits the production of the proinflammatory cytokines such as IL-6, while downregulating the expression of TNF-α, T helper type 1 cytokines, and major histocompatibility complex class 2 molecules (Iyer and Cheng, 2012; Ip et al., 2017). Additionally, IL-10 acts as an immuno-regulator by maintaining homeostasis and preventing host damage during infection (Fournier and Philpott, 2005; Ip et al., 2017).

On a fundamental level, the innate immune system is able to recognize and respond to a wide range of triggers. To do so, it has evolved to include pattern recognition receptors (PRRs) that are able to recognize pathogen-associated molecular patterns (PAMPS) (Chandler and Ernst, 2017). PAMPS are features in microbes that are vital, common, and most importantly, conserved. Present in nearly all bacterial cell walls, peptidoglycan (PepG) is essential for maintaining cell structure and shape (Hergott et al., 2016). PepG protects cells from bursting due to turgor or in response to environmental stressors. The lipid components of cell membranes also contain distinguishing markers that are recognizable and stimulatory. Lipoteichoic acid (LTA), a hallmark of gram-positive bacteria, consists of a glycolipid covalently bound to a hydrophilic glycerophosphate polymer. The glycolipid component localizes in the lipid bilayer of cell membranes. Both PepG and LTA are recognized by the innate immune system and can trigger the systemic release of cytokines (Hergott et al., 2016). When injected into rats during an *in vivo* study, PepG and LTA acted in conjunction to trigger the release of TNF-α (De Kimpe et al., 1995).

A primary designation in the classification of gram-negative bacteria is the presence of lipopolysaccharide (LPS), instead of LTA, as part of the outer cell membrane. LPS, also known as endotoxin, is one of the most potent immunostimulants (Alexander and Rietschel, 2001) and consists of a glycan polymer, oligosaccharide core, and membrane-anchor lipid (Alexander and Rietschel, 2001; Cavaillon, 2018). Physiological recognition of the lipid component by the immune system causes pro-inflammatory cytokine activity (Alexander and Rietschel, 2001; Liu et al., 2018; Monguió-Tortajada et al., 2018; Tawfik et al., 2020). Endotoxin is the main driver of pro- and anti-inflammatory cytokine activation in gram-negative bacterial infections (Feezor et al., 2003; Tawfik et al., 2020). Endotoxin-induced inflammatory conditions cause similar levels of cytokine expression as those found in septic conditions due to a gram-positive bacteria such as *S. aureus*, one of the most common bacteria found on the surface of human skin (Foster, 2004; Tawfik et al., 2020). *S. aureus*, characterized by its thick, PepG layer and LTA layer, is known to stimulate the immune system through the release of toxins into the bloodstream (Feezor et al., 2003; McNicholas et al., 2014). LTA and PepG are the primary sources of activation of cytokines in response to gram-positive bacterial infections (Feuerstein et al., 1994; De Kimpe et al., 1995; Lowings et al., 2009; Cole et al., 2014) whose levels can be predicted using mathematical models.

Mathematical modeling of complex biological systems holds the potential for elucidating emergent properties of intricate biological pathways within the human body (DiLeo et al., 2009; Möbius and Laan, 2015; Morel et al., 2017; Caudill and Lynch, 2018; Meier-Schellersheim et al., 2019). In particular, immune system research can benefit from *in silico* simulations of drug and pathogen responses, which provide a deeper understanding of system dynamics that can be applied to design better diagnostic and treatment protocols (Chow et al., 2005; Eftimie et al., 2016). In general, *in silico* experimentation provides an alternative to tests that are difficult, impractical, expensive, or potentially unethical to perform *in vivo* (Winslow et al., 2012; Nijhout et al., 2015). Additionally, they provide a safer and more cost-effective platform for clinical drug testing, as potential drug candidates can be “pre-screened” (Winslow et al., 2012) Previous simulations successfully modeled activated and resting macrophages, plasma cells, antibodies, helper T cells, T-lymphocytes, and B-lymphocytes in response to the pathogen *S. aureus* (Quintela et al., 2014; Alvarez et al., 2019). However, they failed to account for the interactions between the cellular and cytokine response (Altan-Bonnet and Mukherjee, 2019; Torres et al., 2019; Du and Yuan, 2020).

The mathematical model presented herein, extends previous studies by taking into account the interconnectivity between cellular and cytokine responses and captures *ex vivo* and potential *in vivo* dynamics of the immune response (Quintela et al., 2014; Brady et al., 2016). This study focuses on examining the immune response to acute stimulation by a pathogen, which leads to a cascade of cellular signals recruiting leukocytes, or white blood cells, throughout the body (Hoebe et al., 2004; Anderson et al., 2019; Xue and Falcon, 2019). The cytokines of interest include TNF-*α*, IL-6, IL-8, and IL-10 as they are crucial in downstream signaling pathways that affect inflammatory and other responses within the immune system (Khoa et al., 2003). Additionally, several pharmaceutical agents can influence these cytokines, including Fosfomycin (FOM) (Michalopoulos et al., 2011), Clarithromycin (CAM) (PubMedHealth, 2018), Dexamethasone (DEX), and glucocorticoids (Johnson et al., 2021). FOM is an antibiotic that disrupts the cell walls of bacteria, specifically the gram-positive bacteria *S. aureus*, which blocks the production of IL-8 and amplifies the synthesis of IL-6 and IL-10 (Morikawa et al., 1996; Lan et al., 2018; Zheng et al., 2020). CAM is a macrolide antibiotic that diminishes the spread of bacteria while increasing the production of IL-10 (Morikawa et al., 1996). DEX is a steroid that hinders the immune system in the presence of inflammation (Johnson et al., 2021) by reducing the production of both IL-6 and IL-10 (Morikawa et al., 1996).

The mathematical model representing the basic pathway of the standard human immune response to *S. aureus* (Quintela et al., 2014) was coupled with a mathematical model predicting the cytokine response to LPS (Wang et al., 2000; Brady et al., 2016). Although the two models that were coupled represented responses to different pathogens (gram-positive vs gram-negative bacteria), in both cases the cytokine concentrations are calculated as functions of activated macrophages. Thus, the fundamental interactions between macrophages and cytokines are similar. Several literature sources exploring the immune response to PepG, LTA, and *S. aureus* in conjunction with LPS suggest that our assumption involving both antigens is reasonable (Fournier and Philpott, 2005; Zhang and An, 2007). *S. aureus* was chosen because it is an opportunistic pathogen, and notably is one of the leading causes of life-threatening infections including sepsis (Kwiecinski and Horswill, 2020).

The following mathematical model is formulated using a system of ordinary differential equations (ODEs) and partial differential equations (PDEs) emulating the intricate relationships among the pathogen, cytokines, and cells within the human immune system. As described in detail below, PDEs are used to represent the spatial features of the cellular model, while ODEs are used to model the cytokine activation dynamics. This model is used to simulate the cellular and cytokine activation induced by a low dose of *S. aureus.* Results show the proposed cellular-cytokine mathematical model can be used to predict *ex vivo* and *in vivo* experimental data induced by a given pathogen.

## 2 Methods

### 2.1 Experimental Data

Our coupled mathematical models are validated using data from two studies. The first measured cytokine concentrations from whole human blood induced by *S. aureus* PAMPs in healthy males and the second measured cytokine expression from human endothelial cells removed and induced by a low dose of *S. aureus.* Experiment descriptions are further referenced in **Table 1**.

**Table 1:** Experimental data used in the formulation and validation of the mathematical models.

Experiment Section	Description	Reference
2.1.1	Uses whole human blood data collected from healthy volunteers. The PepG was isolated from *S. aureus*, purified with hydrofluoric acid, and stored at -20°C. Venous blood from healthy subjects was anticoagulated with Na-citrate and analyzed after removal from incubation in the presence or absence of either 10 *μg* of PepG or 100 *μg* of LTA per ml of blood at the 1, 3, 6, 12, and 24-hour mark. The plasma removed during those time periods was centrifuged at 7,000 × *g* for 2 min and stored at -20°C for analyses using the enzyme immunoassay specific to TNF-α, IL-6, and IL-10.	Wang et al., 2000
2.1.2	Endothelial cells (EC) from the human umbilical vein were removed and kept in a 5.5% CO_2_ tissue culture. The ECs were analyzed after removal from incubation in the presence of 10^8^ CFU of *S. aureus* per ml at the 1, 3, 6, 12, and 24-hour mark. The ECs were removed from incubation and centrifuged for 30 min for analysis using enzyme-linked immunosorbent assay (ELISA) to measure IL-8 protein levels in the infected samples.	Yao et al., 1996
2.1.3	Each participant was under EKG signal supervision during the entire experiment. The experiment was initiated with an injection of a low dose (2 *ng/kg* body weight) of LPS. Blood samples were taken before the LPS injections, then at t=2, 3, 3.5, 4, 5, 6, 7, and 8 hours. The experimental protocol was repeated twice, and to reduce the risk of tolerance towards the endotoxin, the two days of experimentation were spaced 4 weeks apart. Following each experimentation day, samples were collected in EDTA tubes (Greiner bio-one, Germany), centrifuged at 4⁰C at 3500 rpm, then analyzed for cytokine concentrations using ELISA. The data from the participants were then analyzed to understand the effects of changes in cytokine expression, particularly TNF-α, IL-6, IL-8 and IL-10.	Brady et al., 2016

#### 2.1.1 Human Whole Blood *Ex Vivo* Response to *S. aureus* PAMPs

We first incorporated data from *ex vivo* studies predicting the response to *S. aureus* PAMPs. This study used whole blood data collected from healthy volunteers (Wang et al., 2000). The PepG was isolated from *S. aureus*, purified with hydrofluoric acid, and stored at -20°C. Venous blood from healthy subjects was anticoagulated with Na-citrate and analyzed after removal from incubation in the presence or absence of either 10 *μg* of PepG or 100 *μg* of LTA per ml of blood at the 1, 3, 6, 12, and 24-hour mark. The plasma removed during those time periods was centrifuged at 7,000 × *g* for 2 min and stored at -20°C for analyses using the enzyme immunoassay specific to TNF-*α*, IL-6, and IL-10 (Wang et al., 2000).

#### 2.1.2 Human Endothelial Cells *Ex Vivo* Response to *S. aureus*

In the second study, endothelial cells (EC) from the human umbilical vein were removed and kept in a 5.5% CO_2_ tissue culture. The ECs were analyzed after removal from incubation in the presence of 10^8^ CFU of *S. aureus* per ml at the 1, 3, 6, 12, and 24-hour mark. The ECs were removed from incubation and centrifuged for 30 min for analysis using enzyme-linked immunosorbent assay (ELISA) to measure IL-8 protein levels in the infected samples (Yao et al., 1996).

The experimental data were collected to formulate cellular-cytokine mathematical models. However, the *ex vivo* experimental data for each cytokine were measured in different units. The whole blood experiments for TNF-*α*, IL-6, and IL-10 were measured in relative concentrations whereas the endothelial cells from an umbilical vein for IL-8 were measured in *ng/ml*. The endotoxin cytokine-based model experiments for all aforementioned cytokines were measured in *pg/ml*. As a result, a conversion factor was implemented to compare the cytokines both to each other and to the simulation model results. This conversion factor used a relative unit metric to the peak value of the 24-hour data. A time-over-time evaluation of the minimum value was divided by the difference between the maximum and minimum values of that instance.


(1) $$relative\;concentration=\frac{\min{\left([cytokine]\right)}_{t}}{\max{\left([cytokine]\right)}_{t}-\min{\left([cytokine]\right)}_{t}}$$

To ensure proper coupling, the cytokine mathematical expressions were initially derived from cytokine concentrations in healthy males induced with low doses of LPS.

#### 2.1.3 Human Cytokine levels *In Vivo* Response to LPS

Cytokine data were extracted from previous studies, approved by the Regional Committee on Health Research Ethics (protocol ID H-3-2012-011) and the Regional Data Monitoring board (ID j-2007-58-0015, local 30-0766), and reported to clinicaltrials.gov (NCT01592526) (Brady et al., 2016). The participants were recruited by means of public advertising in Copenhagen, Denmark, and were required to meet specific safety protocols for the study. The study used data from 20 healthy adult males aged 18-35 years. The inclusion criteria were male, age 18-35, good general health, body mass index < 30 *kg/m^2^*, and written informed consent to participate in the study. The exclusion criteria were daily medicine intake, smoking, allergic reaction to nicotine, and splenectomy.

Each participant was under EKG signal supervision during the entire experiment. The experiment was initiated with an injection of a low dose (2 *ng/kg* body weight) of LPS. Blood samples were taken before the LPS injections, then at t=2, 3, 3.5, 4, 5, 6, 7, and 8 hours. The experimental protocol was repeated twice, and to reduce the risk of tolerance towards the endotoxin, the two days of experimentation were spaced 4 weeks apart. Following each experimentation day, samples were collected in EDTA tubes (Greiner bio-one, Germany), centrifuged at 4⁰C at 3500 rpm, then analyzed for cytokine concentrations using ELISA (Brady et al., 2016). The data from the participants were then analyzed to understand the effects of changes in cytokine expression. This *in vivo* data measuring the cytokine-cytokine response was used to test the differential equation models (Quintela et al., 2014; Brady et al., 2016).

### 2.2 Mathematical Model

**Figure 1:**
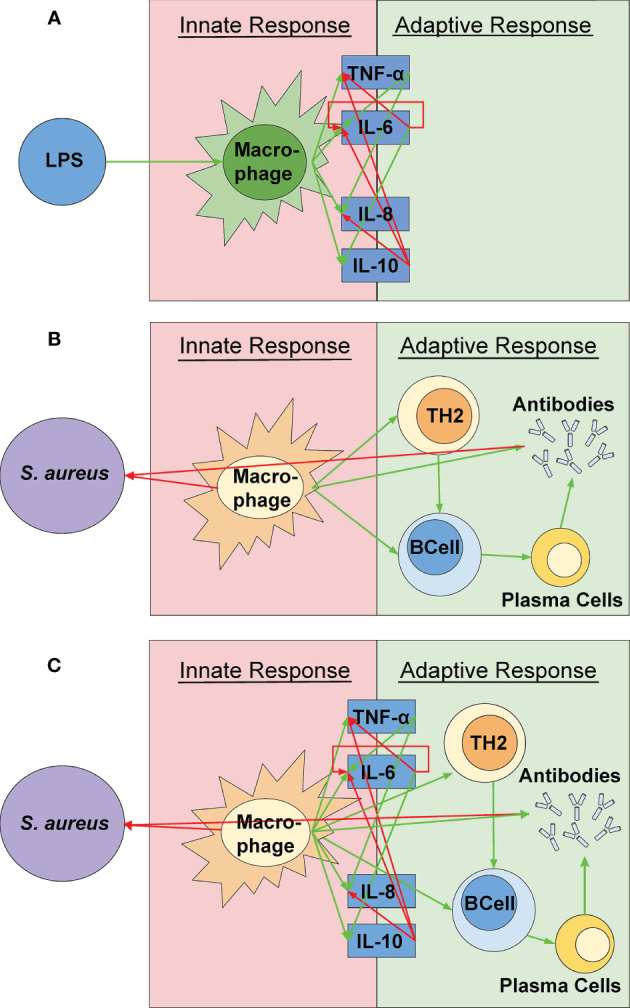
A visual representation of the model components. **(A)** The simulation created by Quintela et al. representing the relationships between *S. aureus* and the different immune response cells were incorporated (Quintela et al., 2014). **(B)** A visual representation of the cytokine mathematical model as outlined by Brady et al. (2016). These relationships were used in the simulation *via* the relationships between the cytokines and the active macrophages rather than the pathogen itself (See **Figure 2C**). **(C)** The coupled model represented in this study. The green and red arrows indicate a positive (up-regulation) and negative (down-regulation) response, respectively. The various shapes indicate the parts of the simulation from Quintela et al. while the dark blue boxes indicate the parts of the simulation from the mathematical equations from Brady et al. The concentrations of the cytokines are solely dependent on the macrophage concentration and are not directly affected by the pathogen itself.

A novel cellular-cytokine mathematical model (shown in **Figure 1**) combining the cellular model by Quintela et al. and the inflammatory cytokine model by Brady et al. is proposed (Quintela et al., 2014; Brady et al., 2016). The cellular model by Quintela et al. predicts the activation of the acquired immune response by activated macrophages acting as antigen presenting cells to the bacterium *S. aureus*, present in a portion of lung tissue. The prediction of resting and activated macrophages is used as an input to the novel cellular-cytokine model predicting the cytokine response to a pathogen. The novel cellular-cytokine model described by a system of partial (PDEs) and ordinary (ODEs) differential equations is solved over a 24-hour period. The cellular model is first solved in C++ and then used as input for the novel cellular-cytokine model, which ODEs are solved in MATLAB, and predicted states are compared to the experimental data for cellular and cytokine concentrations in response to bacterial infection with *S. aureus.*

As noted in the introduction, numerous research studies have concluded that the host response to gram-positive and gram-negative bacteria provides similar activation of pro- and anti-inflammatory cytokines (Feezor et al., 2003; Fournier and Philpott, 2005). In an *ex vivo* study of 52 healthy patients, Feezor et al. concluded that activation of TNF-⍺, IL-8, and IL-10 induced by LPS and *S. aureus* show no significant difference in cellular activation and cytokine expression. This is further supported in studies by De Kimpe et al., 1995; Feezor et al., 2003; Fournier and Philpott, 2005, finding that LTA and PepG promoting activation of pro-inflammatory, anti-inflammatory, and chemoattractant properties have similar effects as a host response to LPS. As a result, the combination of LPS from gram-negative bacteria and PAMPs from gram-positive bacteria is reasonable due to their ability to induce similar inflammatory responses (Fan et al., 2007).

The widespread inflammation and septic shock caused by gram-positive bacteria, such as *S. aureus*, is primarily due to the function of LTA and PepG during an inflammatory response (Feezor et al., 2003; Moreillon and Majcherczyk, 2003; Fournier and Philpott, 2005). LTA and PepG work in conjunction to induce cytokine expression in the host’s innate and adaptive immune response to *S. aureus*. Mechanisms of action in the host including phagocytosis, neutrophil flux, and Sbi protein activation differ between the wall components and the pathogen itself, but they elicit similar inflammatory cytokine responses (Feezor et al., 2003; Moreillon and Majcherczyk, 2003; Fournier and Philpott, 2005). Results from these studies promote our assumption that the inflammatory response to the gram-positive bacteria *S. aureus*, the gram-positive bacteria cell wall constituents LTA and PepG, and the gram-negative bacteria wall constituent LPS, induce similar cytokine expression *ex vivo* (Feezor et al., 2003; Moreillon and Majcherczyk, 2003; Fournier and Philpott, 2005) but differ in their mechanism of action and host recognition.

These studies serve as a foundation for our coupled mathematical model combining the cellular PDEs and the cytokine ODEs *via* a shared variable, representing the concentration of activated macrophages averaged over the inflamed tissue.

#### 2.2.1 Cellular Model

The cellular model by Quintela et al. predicts the activation of the acquired immune response to *s. aureus* as a function of the concentrations of bacteria (*A*), resting (M) and activated macrophages (*M*), and antibodies (*F*) spatially distributed in 1cm^3^ of lung tissue (*x* = (*x,y,z*)) as well as concentrations of lymphocytes (*T,B*), plasma cells (*p*), and antibodies (*F*) varying in time at the nearest lymph node. In this study, we assume that the the activated macrophages act as antigen presenting cells and migrate to the nearest lymph node where the specific response is triggered. Specific antibodies then travel to the infection site to opsonize the antigen. The activated macrophages are modelled both as spatially interacting with the antigen in the tissue (*M_A_* (*x,t*)) and at the lymph node $\left(M_{A}^{L}(t)\right)$ interacting with the lymphocytes with concentrations varying only in time. The model is formulated using a system of partial and ordinary differential equations with the following dependent variables:

Spatial variables (*pg/mm^3^*):

- *S. aureus* bacteria (*A*(*x,t*))
- Resting Macrophages (*M_R_* (*x,t*))
- Activated Macrophages (*M_A_* (*x,t*))
- Specific Antibodies (*F*(*x,t*))

Temporal variables (*pg/mm^3^*):

Average Activated Macrophages $\left(M_{A}^{L}(t)\right)$

- T-Lymphocytes (*T*(*t*))
- B-Lymphocytes (*B*(*t*))
- Plasma cells (*P*(*t*))
- Antibodies (*F^L^* (*t*))

The details of the cellular model equations and the coupling from the tissue and nearest lymph node are available in Quintela et al. (2014). The novel cellular-cytokine model that is based both on the macrophage activation part of this cellular model and on the inflammatory cytokine model by Brady et al, is described below.

#### 2.2.2 Novel Cellular-Cytokine Model

The cellular and cytokine dynamics are described using ODEs. We predict concentrations of TNF-*α*, IL-6, IL-8, and IL-10 as a function of the resting (*M_R_* (*t*)) and activated macrophages (*M_A_* (*t*)) considering the average tissue concentration obtained from the cellular model as initial condition. This model was originally developed to study the response of cytokines to LPS, but since LPS and PAMPs induce similar pro- and anti-inflammatory responses, we couple the cytokine model to the cell-based model described in Section 2.2.1. This is supported by findings from Fan et al. who discovered that *Gi* proteins present in both gram-negative and gram-positive bacteria contribute to the regulation of several cytokines and chemokines in response to bacterial stimuli (Fan et al., 2007). In the cytokine model, the dependent variables include:

Temporal variables:

- Bacteria: *A*(*t*)
- Resting Macrophages: *M_R_* (*t*)
- Activated Macrophages: *M_A_* (*t*)
- Tumor Necrosis Factor *α* (pro-inflammatory): *TNF*(*t*)
- Interleukin-6 (pro-inflammatory): : *IL6*(*t*)
- Interleukin-8 (pro-inflammatory): : *IL8*(*t*)
- Interleukin-10 (anti-inflammatory): *IL10*(*t*)

For each cytokine, up-and down-regulation is modeled using sigmoidal functions given by


(2) $$H_{Y}^{U}(X)=\frac{X^{h}}{\eta_{YX}^{h}+X^{h}}or\;H_{Y}^{D}(X)=\frac{\eta_{YX}^{h}}{\eta_{YX}^{h}+X^{h}},$$

where X represents the cytokine inducing up-regulation (superscript U) or down-regulation (superscript D) of cytokine Y. The half-maximum value is represented by *η*. These sigmoidal functions are incorporated within all the cytokine equations to describe the relationships between each of the cytokines. Specific parameter values are given in **Table 2**.

**Table 2:** Parameters, values, and units for the variables in the partial and ordinary differential equations found in the simulation (6, 7).

Parameter	Value	Unit	Reference
*k_MTNF_*	8.65	*hr* ^-1^	Brady et al., 2016
*k_TNF_*	200	*day* ^–1^	Brady et al., 2016
*k_TNFM_*	1.5	$\frac{relative\;cytokine\;concentration}{day\cdot \#\;of\;cells}$	Brady et al., 2016
*k_6_*	4.64	*day* ^-1^	Brady et al., 2016
*k_6M_*	0.01	$\frac{relative\;cytokine\;concentration}{day\cdot \#\;of\;cells}$	Brady et al., 2016
*k_6TNF_*	0.81	$\frac{relative\;cytokine\;concentration}{day\cdot \#\;of\;cells}$	Brady et al., 2016
*k_8_*	0.464	*day* ^-1^	Brady et al., 2016
*k_8M_*	0.056	$\frac{relative\;cytokine\;concentration}{day\cdot \#\;of\;cells}$	Brady et al., 2016
*k_8TNF_*	0.56	$\frac{relative\;cytokine\;concentration}{day\cdot \#\;of\;cells}$	Brady et al., 2016
*k_10_*	1.1	*day* ^-1^	Brady et al., 2016
*k_10M_*	0.19	$\frac{relative\;cytokine\;concentration}{day\cdot \#\;of\;cells}$	Brady et al., 2016
*k_106_*	0.0191	$\frac{relative\;cytokine\;concentration}{day\cdot \#\;of\;cells}$	Brady et al., 2016
*q_TNF_*	0.14	*relative concentration*	Brady et al., 2016
*q_IL6_*	0.6	*relative concentration*	Brady et al., 2016
*q_IL8_*	0.2	*relative concentration*	Brady et al., 2016
*q_IL10_*	0.15	*relative concentration*	Brady et al., 2016
*η_TNF6_*	560	*relative concentration*	Brady et al., 2016
*η_TNF10_*	17.4	*relative concentration*	Brady et al., 2016
*η_610_*	34.8	*relative concentration*	Brady et al., 2016
*η_66_*	560	*relative concentration*	Brady et al., 2016
*η_6TNF_*	185	*relative concentration*	Brady et al., 2016
*η_810_*	17.4	*relative concentration*	Brady et al., 2016
*η_8TNF_*	185	*relative concentration*	Brady et al., 2016
*η_106_*	560	*relative concentration*	Brady et al., 2016
*h_106_*	3.68	*dimentionless*	Brady et al., 2016
*h_6TNF_*	2	*dimentionless*	Brady et al., 2016
*h_66_*	1	*dimentionless*	Brady et al., 2016
*h_610_*	4	*dimentionless*	Brady et al., 2016
*h_8TNF_*	3	*dimentionless*	Brady et al., 2016
*h_810_*	1.5	*dimentionless*	Brady et al., 2016
*h_TNF10_*	3	*dimentionless*	Brady et al., 2016
*h_TNF6_*	2	*dimentionless*	Brady et al., 2016
*D_A_*	3.7·10^-15^	$\frac{mm^{3}}{day}$	Quintela et al., 2014
*D_MR_*	4.32·10^-2^	$\frac{mm^{3}}{day}$	Quintela et al., 2014
*D_MA_*	0.3	$\frac{mm^{3}}{day}$	Quintela et al., 2014
*β_A_*	2.0	*day* ^-1^	Quintela et al., 2014
*k_A_*	50.0	$\frac{cell}{mm^{3}}$	Quintela et al., 2014
*μ_A_*	0.1	*day* ^–1^	Quintela et al., 2014
*μ_MR_*	0.033	*day* ^-1^	Quintela et al., 2014
*μ_MA_*	0.07	*day* ^-1^	Quintela et al., 2014
*γ_MA_*	8.3·10^-2^	$\frac{mm^{3}}{cell\cdot day}$	Quintela et al., 2014
*λ_MR_*	5.98·10^-3^	$\frac{mm^{3}}{cell\cdot day}$	Quintela et al., 2014
*λ_MA_*	5.98·10^-2^	$\frac{mm^{3}}{cell\cdot day}$	Quintela et al., 2014
*λ_AFMR_*	1.66·10^-3^	$\frac{mm^{6}}{cell^{2}\cdot day}$	Quintela et al., 2014
*λ_AFMA_*	7.14·10^-2^	$\frac{mm^{6}}{cell^{2}\cdot day}$	Quintela et al., 2014
*α_MA_*	10^-3^	*day* ^-1^	Quintela et al., 2014
*α_MR_*	4.0	*day* ^-1^	Quintela et al., 2014

These parameters can be adjusted to depict in vivo conditions.

The cellular portion of the model proposed herein, predicts concentrations (*pg/mm^3^*) of bacteria (*A*), resting (*M_R_*) and activated macrophages (*M_A_*) varying over time.

*S. aureus* bacterium (*A*(*x, t*)) growth rate and rate of decline is modeled as:


(3) $$\begin{array}{c} \frac{dA}{dt}=\beta_{A}A\left(1-\frac{A}{k_{A}}\right)-\mu_{A}A-\lambda_{MR}M_{R}A-\lambda_{MA}AM_{A}\\ A(0)=A_{0},\\ A(x,0)=A_{0},\;\frac{dA}{dt}(\cdot ,t){|}_{d\Omega }=0 \end{array}$$

where the first term represents the logistic growth of the bacteria, the constant *k_A_* represents the carrying capacity, and *β_A_* the replication rate. The second term gives the natural decay rate of the bacteria in the absence of any immune system processes through the natural decay coefficient, μ_A_. The third and fourth terms describe the phagocytosis of *S. aureus* through activated and resting macrophages, with the constants *λ_MA_* and *λ_MR_* representing the rate of decline caused by activated and resting macrophages, respectively.

Resting *M_R_* (*t*) macrophages response to the pathogen *S. aureus* are modeled as:


(4) $$\begin{array}{c} \frac{dM_{R}}{dt}=\mu_{MR}\left(1-\frac{M_{R}}{M_{R\_MAX}}\right)M_{R}-(\gamma_{MA}+k_{MTNF}H_{M}^{U}(TNF)H_{M}^{D}(IL10))M_{R}A\\ M_{R}(0)=\bar{M_{R}}, \end{array}$$

where the first term in Eq (3) represents the constant influx rate of the resting macrophage (*μ_MR_* up to *M_R MAX_* and the second term represents macrophage activation at the rate *γ_MA_* in response to the bacteria and activation rate *k_MTNF_* considering the influence of the cytokines TNF and IL-10. The initial condition is given by the average of resting macrophages in the tissue $\left({\bar{M}}_{R}\right)$ as an outcome of the cellular model simulation over 24h.

Activated macrophages *M_A_* (*t*) are modeled as:


(5) $$\begin{array}{c} \frac{dM_{A}}{dt}=(\gamma_{MA}+k_{MTNF}H_{M}^{U}(TNF)H_{M}^{D}(IL10))M_{R}A-\mu_{MA}M_{A},\\ M_{A}(0)=\bar{M_{R}}, \end{array}$$

where the first term in Eq (4) represents macrophage activation at the rate *γ_MA_* and *k_MTNF_* considering the influence of the cytokines TNF and IL-10, and the last term represents the decay rate of the activated (*μ_MA_*) macrophages. The initial condition is given by the average of activated macrophages in the tissue $\left({\bar{M}}_{A}\right)$ as an outcome of the cellular model simulation over 24h. The initial concentration of both resting and activated macrophages are constant $\left(M_{R_{0}}\;and\;M_{A_{0}}\right)$ and at the boundary of the tissue neither the resting or activated macrophage concentration change.

In response to macrophage activation, the Tumor Necrosis Factor alpha (TNF-*α*) dynamics can be modeled as:


(6) $$\frac{dTNF}{dt}=k_{TNFM}H_{TNF}^{D}(IL6)H_{TNF}^{D}(IL10)M_{A}-k_{TNF}(TNF-q_{TNF}),$$

where the first term represents the down-regulating interactions that the cytokines IL-6 and IL-10 have with TNF-⍺ growth (at rate *k_TNFM_*) mediated by the average concentration of activated macrophages. The second term describes the rate in which TNF-⍺ naturally decays over time. As noted in the equation, the rate of change of TNF-*α* depends on the activated macrophages, which is predicted from the cellular model. In the cellular model, *M_A_* depends on both *x* and *t*. This response is integrated here and described further in Section 2.2.3.

Interleukin 6 (IL-6) activation is modeled as:


(7) $$\frac{dIL6}{dt}=(k_{IL6M}+k_{IL6TNF}H_{IL6}^{U}(TNF))H_{IL6}^{D}(IL6)H_{IL6}^{D}(IL10)M_{A}-k_{IL6}(IL6-q_{IL6}),$$

where the first term represents the interactions between TNF-⍺ (upregulating) and IL-10 (downregulating) affecting IL-6 production at a rate (*k_IL_*_6_ *_TNF_*), and an IL-6, which also induce auto-negative feedback. The second term represents the natural decay (at rate *k*_*IL*6) of IL-6 towards a resting level (*q_IL_*_6_).

Interleukin 8 (IL-8) activation is modeled as:


(8) $$\frac{dIL8}{dt}=(k_{IL8M}+k_{IL8TNF}H_{IL8}^{U}(TNF))H_{IL8}^{D}(IL10)M_{A}-k_{IL8}(IL8-q_{IL8}),$$

where the first term represents the interactions between the opposing effects of TNF-⍺ (upregulating) at a rate (*k_IL8TNF_*) and IL-10 (downregulating) at a rate (*k_IL_*_8_ *_M_*) stimulating the growth of IL-8 at a rate proportional to the average concentration of activated macrophages production, while the second term represents the natural decay rate of IL-8.

Interleukin 10 (IL-10) activation is modeled as:


(9) $$\frac{dIL10}{dt}=(k_{IL10M}+k_{IL10IL6}H_{IL10}^{U}(IL6))M_{A}-k_{IL10}(IL10-q_{IL10}),$$

where the first term describes the up-regulation of IL-10 due to IL-6 (at a rate *k_IL_*_10_ *_IL_*_6_) and average concentration of activated macrophages (at a rate *k_IL_*_10_ *_M_*), while the second term describes the natural decay rate of IL-10.

#### 2.2.3 Coupled Model Numerical Solution

We first solve the cellular spatiotemporal model (over a 24-hour period) predicting the average concentrations of resting and activated macrophages in response to a low dose of *S. aureus* in the tissue. It is assumed that pathogen and macrophage movements can be represented as diffusion according to Fick’s Law (Crank, 1975). Therefore, each PDEs include a diffusion term with a specific diffusivity coefficient (*D_i_*, = *MR*, *MA*) estimated by Quintela et al. based on experimental observations for the pathogen and the cells that are included in this study. The diffusion term representing the rate of transfer of cells from one site to another is proportional to their concentration gradient. For simplicity, it is assumed that the medium is isotropic and has the same diffusion coefficient for every direction (Quintela et al., 2014), i.e., we model diffusion *via* the term, *D_i_* Δ*M_j_*, *i* = *MR*, *MA* and *j*= *R*, *A* denote the diffusion of the macrophages in the tissue, again Δ refer to the second order derivative in space. At the onset of the simulation there aren’t any activated macrophages in the tissue (*MA*_0_ = 0), i.e., the resting macrophages that are equally distributed over the domain at concentration *MR*_0_. The cellular model is solved in C++ using the finite differences method.

Following the simulation of the cellular model, we calculate the average number of resting and activated macrophages by integrating the resulting concentrations of each over the discretized 1 cubic cm domain as:


(10) $$\bar{M}(t)=\frac{1}{V}\int_{\Omega }M\;d\;\Omega$$

The average concentrations for resting ${\bar{M}}_{R}$ , and activated ${\bar{M}}_{R}$ , macrophages are used as initial condition for the novel cellular-cytokine model. The ODEs ((1) - (8)) are then solved in MATLAB using the ode45 function.

### 2.3 Model Parameters

The model parameters include diffusion coefficients and replication, decay, activation, phagocytic, and migration rates of bacteria and cellular macrophages from the cellular model, and cytokine rate constants, source terms, and half-maximum values exponents from the cytokine model. To simplify the complexity of the human immune response, the cellular model parameters, cytokine half-maximum value and Hill function exponent parameters are held constant. They can be adjusted for model specifications, including representing *in vivo* septic conditions.

*Diffusion coefficients: D_i_* denotes the diffusion of the particular bacteria or cell into the tissue. Values from all cellular model parameters were obtained from literature research (Marchuk, 1997; Sarah and Richard, 2009; Pigozzo et al., 2013).

*D_A_* - Bacteria diffusion coefficient
*D_MR_* - Resting macrophage diffusion coefficient
*D_MA_* - Activated macrophage diffusion coefficient

*Replication, decay, activation, and phagocytic rates:* The following parameters denote the different rates of growth and decay for the bacteria and cells.

*β_A_* - Replication rate of the bacteria
*k_A_* - Carrying capacity of the bacteria
*μ_A_* - Natural decay rate of the bacteria
*μ_MR_* - Natural decay rate of the resting macrophages
*μ_MA_* - Natural decay rate of the activated macrophages
*γ_MA_* - Rate in which resting macrophages become active
*λ_MR_* - Activation of the macrophages
*λ_MA_* - Destruction rate of the bacteria by activate macrophages
*λ_AF│MR_*- Destruction rate of opsonized bacteria by resting macrophages
*λ_AF│MA_* - Destruction rate of opsonized bacteria by activated macrophages

*Cellular rate of migration:* The *α_i_* denotes the migration rate of the macrophages to the site of infection.

*α_MA_* - Migration rate of activated macrophages
*α_MR_* - Migration rate of resting macrophages

*Rate constants: k_i_* denotes cytokine activation or elimination rates, and *k_ij_* determines the rate of change in the upregulation rate of a cytokine secreted from activated macrophages. These parameters have initial values based on predicted conditions of our model activated with a low dose of *S. aureus*.

*k_MTNF_* – Activation rate of resting macrophages influenced by TNF-α
*k_TNF_* - Activation rate (per hour) of TNF-α
*k_TNFM_*- Upregulation of TNF-α by the activated macrophages
*k_6_*- Activation rate (per hour) of IL-6
*k_6M_*- Upregulation of IL-6 by the activated macrophages
*k_6TNF_*- Upregulation of IL-6 by TNF-α
*k_8_*- Activation rate (per hour) of IL-8
*k_8M_*- Upregulation of IL-8 by the activated macrophages
*k_8TNF_* - Upregulation of IL-8 by TNF-α
*k_10_* - Activation rate (per hour) of IL-10
*k_10M_* - Upregulation of IL-10 by the activated macrophages
*k_106_* - Upregulation of IL-10 by IL-6

*Source parameters*: *q_i_* represents the base concentration of each cytokine in the absence of pathogen stimulation. *q_i_* are also used to set initial conditions for each cytokine. These parameters values are established based on initial predicted conditions of the model in the absence of *S. aureus*.

*q_TNF_* - The concentration of TNF-α in the absence of a pathogen
*q_IL6_* - The concentration of IL-6 in the absence of a pathogen
*q_IL8_* - The concentration of IL-8 in the absence of a pathogen
*q_IL10_* - The concentration of IL-10 in the absence of a pathogen

*Half-maximum value:* The *η_i_* parameters describe the effector cytokine concentration at which target cytokine activity would reach half-maximum with units of *pg mL^-1^*. They are included in sigmoidal Hill functions used to model up- or down-regulation of a specific target cytokine by a specific effector cytokine.

*η_TNF6_* - Half-maximum value associated with downregulation of TNF-α by IL-6
*η_TNF10_* - Half-maximum value associated with downregulation of TNF-α by IL-10
*η_610_* - Half-maximum value associated with downregulation of IL-6 by IL-10
*η_66_*- Half-maximum value associated with the auto-negative feedback of IL-6
*η_6TNF_* - Half-maximum value associated with upregulation of IL-6 by TNF-α
*η_810_* - Half-maximum value associated with downregulation of IL-8 by IL-10
*η_8TNF_* - Half-maximum value associated with upregulation of IL-8 by TNF-α
*η_106_*- Half-maximum value associated with upregulation of IL-10 by IL-6

*Hill function exponent: ℎ_i_* represents the steepness of the Hill functions used to model the up- or down-regulation of each interaction.

*h_106_*- Hill function exponent associated with upregulation of IL-10 by IL-6
*h_6TNF_* - Hill function exponent associated with upregulation of IL-6 by TNF-α
*h_66_* - Hill function exponent associated with auto-negative feedback of IL-6
*h_610_* - Hill function exponent associated with downregulation of IL-6 by IL-10
*h_8TNF_* - Hill function exponent associated with upregulation of IL-8 by TNF-α
*h_810_*- Hill function exponent associated with downregulation of IL-8 by IL-10
*h_TNF10_*- Hill function exponent associated with downregulation of TNF-α by IL-10
*h_TNF6_* - Hill function exponent associated with downregulation of TNF-α by IL-6

Parameters for half-maximum (*η_i_*) and the respective exponents (*h_i_*) are fixed at their nominal values given in **Table 2**, while the rate constants *k* and source parameters *q* are estimated to fit the model to data.

### 2.4 Sensitivity Analysis

We have performed a sensitivity analysis to estimate how the model solution is affected by small perturbations to each model parameter. The sensitivity index was defined as the ratio


(11) $$S_{i}=\frac{\delta J}{J}\frac{\delta p}{p},J,p\neq 0$$

where *j* denotes a model output that depends on a parameter *p*, *δ* is a perturbation to the parameter *p*, and *δJ* is the resulting perturbation to the output *j*. The sensitivity index is a measure of the percentage of change in the output given a perturbation in each parameter (Quintela et al., 2014). The value of each parameter was varied by 10%, while other parameters were kept fixed at their baseline values.

### 2.5 Parameter Estimation

Parameters for the cell model are taken from literature, whereas we estimate rate constants and source parameters for the cytokine model minimizing the least squared error (*j*) between model predictions and data, given by


(12) $$\begin{array}{ll} J=r^{T}r, & r=\{r_{TNF},r_{IL6},r_{IL8},r_{IL10}\} \end{array}$$

where *r_X_* = {*X^m^* (*t*_1_) – *X^d^* (*t*_1_), *X^m^* (*t*_2_) – *X^d^* (*t*_2_), …, *X^m^* (*t*_N_) – *X^d^* (*t*_N_)}/ $\sqrt{N}$ ,and *X* = *TNF*, *IL*6, *IL*8, *IL*10 Superscript (*m*) refer to the model prediction and superscript (*d*) to the data. Parameter estimation was done using the MATLAB *fminsearch* function.

### 2.6 Statistical Analysis

A regression model was used to compare simulated results to experimental data. In each regression, simulated relative concentrations of each cytokine were compared to experimental concentrations of the same cytokine, induced by either LTA or PepG.

We assume linear regression of the form,


(13) $$y=c_{1}+c_{2}x,$$

Where *c*_1_ represents the y-intercept and *c*_2_ represents the slope of each linear least squares (LLS) regression. This analysis was completed in MATLAB using the linear least squares package *fitlm* and shows the significance of the simulation relative to the experimental data (**Table 3**).

**Table 3:** LLS regressions of simulated results for each cytokine vs. experimental data of immune response to LTA and PepG.

Cytokine- Inducer	Var.	Est. value	Sth. Err.	95% Con. Int.	t-stat	p-val.	RMSE	R^2^	R^2^ Adj.	F-stat vs. Const.	p-val.
TNFα-LTA	*c*1	-11.51	11.85	11.6	-0.97131	0.4339	14.6	0.946	0.919	34.9	0.0275
	*c*2	1.117	0.189	0.185	5.908	0.0275					
TNFα-PepG	*c*1	-1.995	11.187	8.95	-0.178	0.867	16	0.875	0.844	28	0.00612
	*c*2	0.969	0.183	0.15	5.291	0.00612					
IL6-LTA	*c*1	-10.686	7.094	5.68	-1.506	0.206	10.7	0.955	0.944	85.8	0.000756
	*c*2	1.125	0.121	0.10	9.262	0.000756					
IL6-PepG	*c*1	-9.645	6.001	4.80	-1.605	0.184	9.04	0.958	0.948	92.2	0.000657
	*c*2	0.988	0.102	0.08	9.604	0.000657					
IL8- PepG/LTA	*c*1	-10.534	6.512	5.71	-1.618	0.204	9.73	0.96	0.947	72.2	0.00342
	*c*2	1.043	0.123	0.11	8.497	0.00342					
IL10-LTA	*c*1	-13.236	11.900	9.52	-1.112	0.328	18	0.827	0.784	19.1	0.0120
	*c*2	0.923	0.211	0.17	4.371	0.0120					
IL10-PepG	*c*1	-12.852	15.080	12.07	-0.852	0.442	22.8	0.704	0.63	9.52	0.0367
	*c*2	0.825	0.267	0.21	3.086	0.0367					

## 3 Results

The results illustrate the model simulation in comparison to experimental data. Similar to LPS, *S. aureus* causes an initial spike in the concentrations of the cytokines. As the bacteria multiply, cytokine concentrations increase, reaching a plateau at which the bacterial growth rate equals the immune response rate. At this stage, the immune response decreases until the response rate overcomes the growth of the bacteria (Brennan and Zheng, 2007).

**Figure 2:**
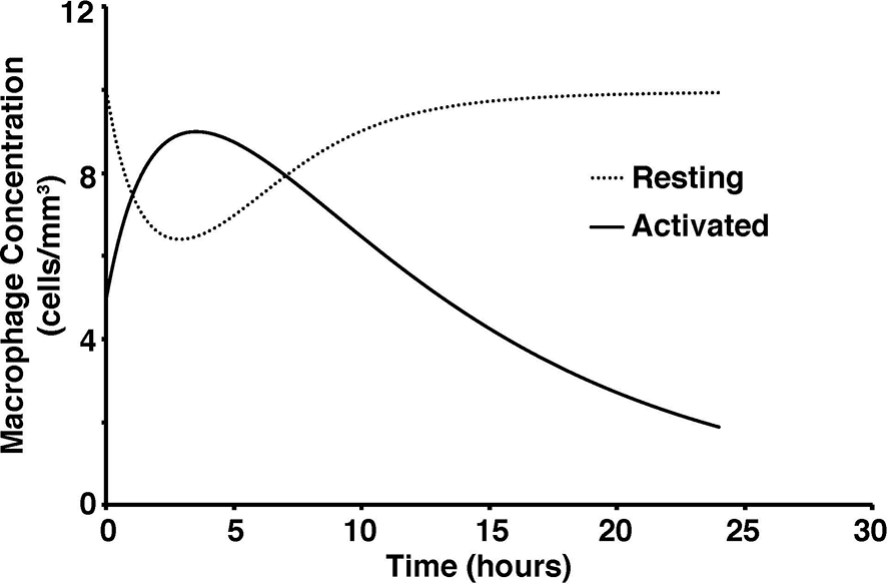
Concentration of activated and resting macrophages over a 24-hour period.

**Figure 3:**
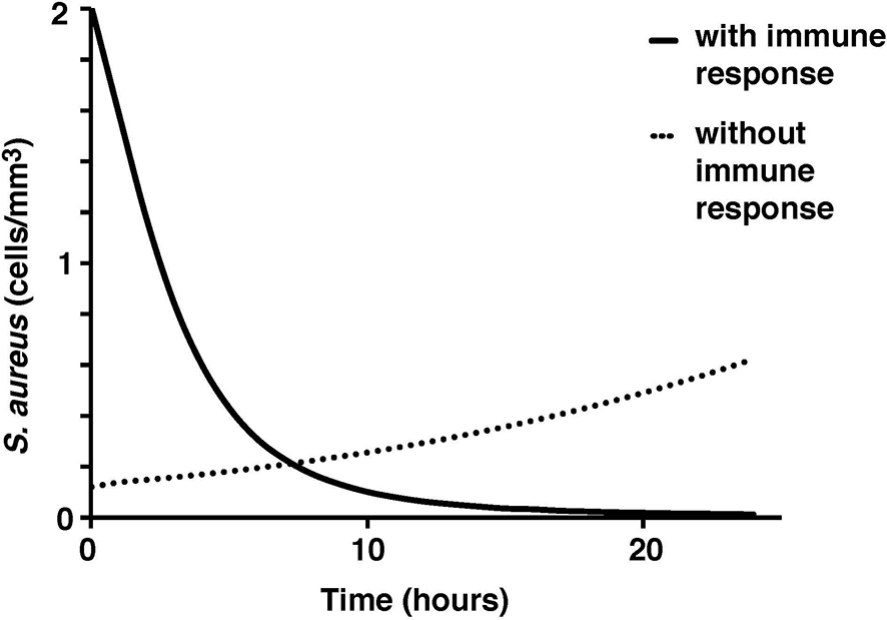
*S. aureus* average cell concentration in the tissue with and without immune response over a 24-hour period.

To test the validity of the model on a macroscale, the model was used to simulate a response to an infection by *S. aureus*. As expected, there were clear indications of the immune system response. From the start of infection, simulated macrophage and cytokine concentrations increase in proportion to the activated macrophages until a maximum concentration is reached at five hours (**Figure 2**). Once the bacteria were eliminated by the immune system response in the simulation, the macrophages and cytokines started reverting back to their basal values (**Figure 3**).

**Figure 4:**
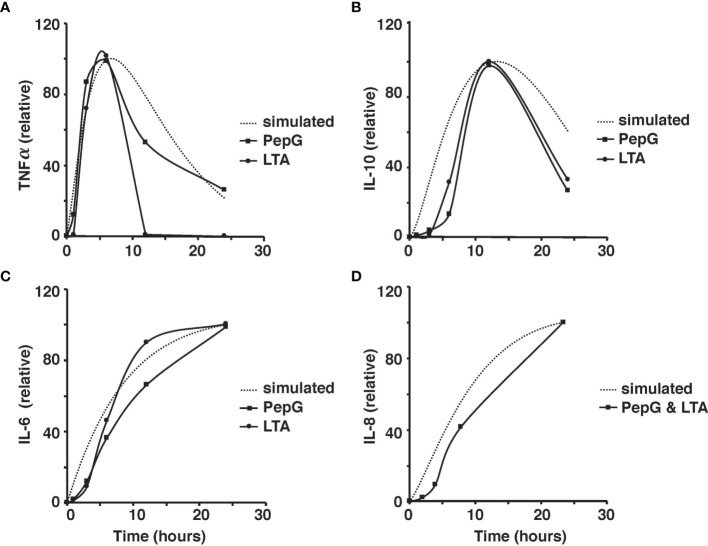
Comparison of the simulated TNF-⍺ and experimental TNF-⍺ activity in response to the introduction of 10 *μg* of PepG/mL or 100 *μg* of LTA/mL of human blood over the 24-hour period **(A)**. Comparison of the simulated IL-6 and experimental IL-6 activity based on the introduction of 10 *μg* of PepG/mL or 100 *μg* of LTA/mL of human blood over a 24-hour period **(B)**. Comparison of the simulated IL-10 and experimental IL-10 activity based on the introduction of 10 microgram of PepG/mL or 100 *μg* of LTA/mL of human blood over the 24-hour period **(C)**. Comparison of the simulated IL-8 and experimental IL-8 activity based on the introduction of *S. aureus*-infected endothelial cells containing 10 *μg* of PepG/mL and 100 *μg* of LTA/mL over the 24-hour period **(D)**. All cytokine concentrations are relative values as discussed in the methods.

The microscale validity of the simulation was tested through close comparison of the simulation to the studies of *ex vivo* stimulation conducted by Wang et al. and Yao et al. (Yao et al., 1996; Wang et al., 2000; Wang and Deisboeck, 2014). Cytokine response by LTA and PepG stimulation in whole blood samples from those studies closely resemble the simulated cytokine response described herein (Yao et al., 1996; Wang et al., 2000). The peaks of TNF-α, IL-10, IL-6, and IL-8 coincide between the model results and *ex vivo* blood sample data (Yao et al., 1996; Wang et al., 2000) (**Figure 4**).

**Figure 5:**
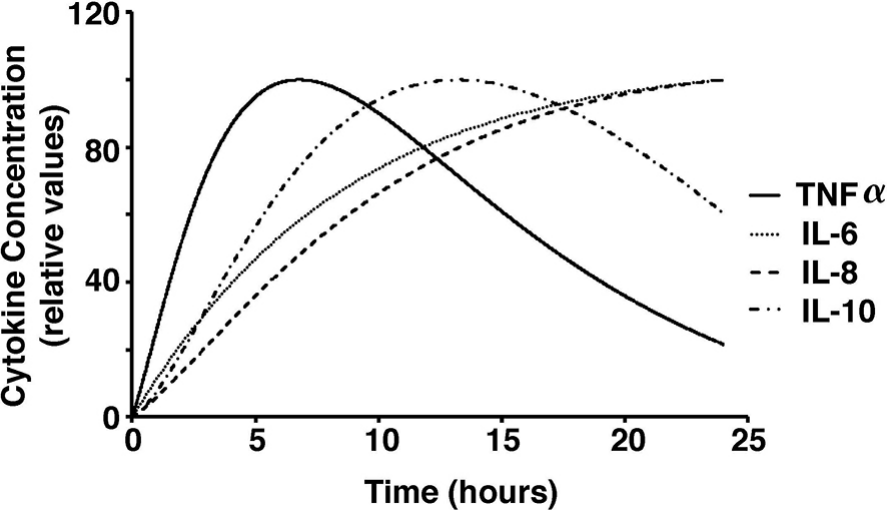
Cytokine concentrations over a 24-hour period in response to stimulations with low dose *S. aureus* in our model.

Due to the different magnitudes of cytokine concentrations in the coupled simulation and blood sample data, a conversion factor to convert concentrations to relative values was introduced for meaningful comparison (Wang et al., 2000). Due to the limitations in the whole blood samples, TNF-⍺, IL-6, IL-8, and IL-10 were studied in response to low concentrations of PepG and LTA. On the contrary, the coupled simulation studied the immune response to antigen concentrations at significantly higher concentrations. Thus, the initial cytokine, antigen, and macrophage concentrations were modified to match the experimental conditions for a more accurate comparison (**Figure 5**).

**Figure 6:**
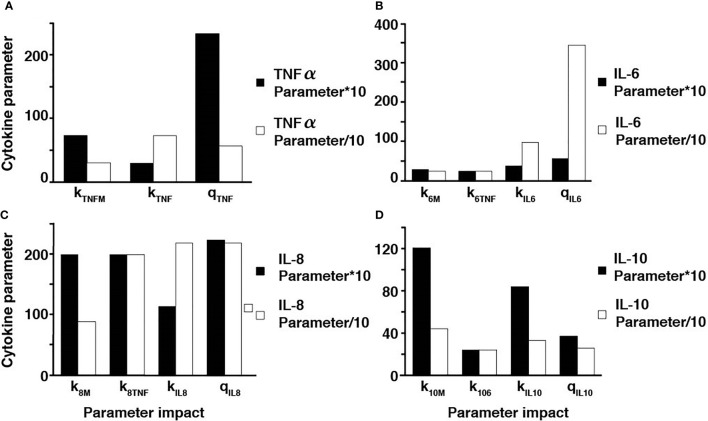
Parameter adjustments of the individual cytokines. A ten-fold increase and decrease in the TNF-⍺ parameters **(A)**, IL-6 parameters **(B)**, IL-8 parameters **(C)**, and IL-10 parameters **(D)**.

**Figure 7:**
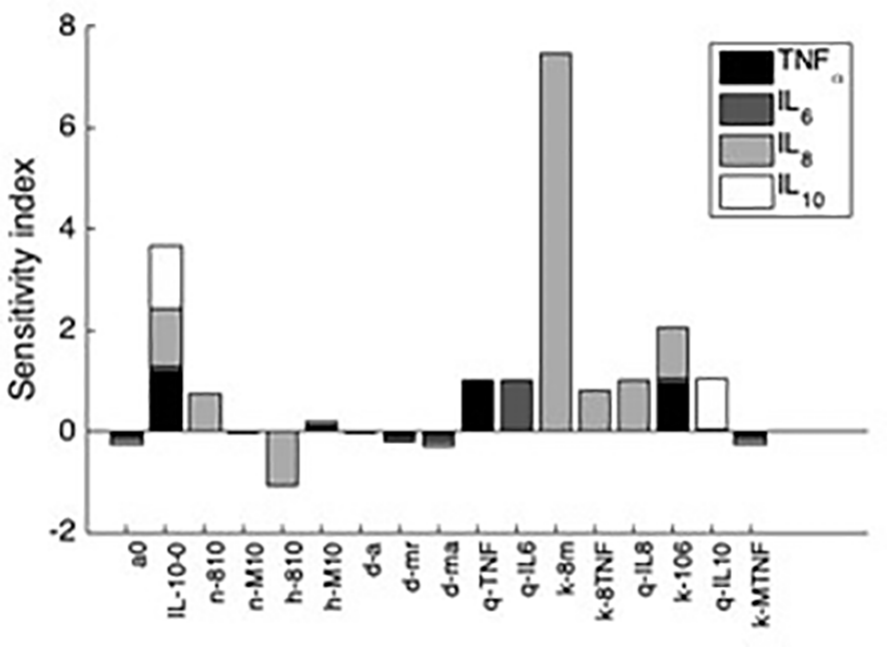
Sensitivity indices denoting the most influential parameters to each cytokine after 24h simulation. Shown are the first 10 parameters that influence at least a 10% change in the resulting value of at least one of the four analyzed variables. Negative sensitivity indexes indicate reduced cytokine output while the omitted parameters trivially affected cytokine output.

Additionally, sensitivity analyses were conducted to validate the *ex vivo* experimental data with the simulated results. Each parameter in the cytokine equations were tested through its real-time concentration changing fold number, acquired by dividing the estimated parameters by their initial concentration and integrated over a 24-hour period (**Figures 4**, **5**). The sensitivity index of 50 parameters in the model following a 24-hour simulation was conducted by implementing 10% parameter variations in each cytokine (**Figure 6**, **7**). The parameters *q_TNF_* and *k_TNF_* in the TNF-⍺ equation, *q_IL_*_8_ and *k*_8_ in the IL-8 equation, *q_IL_*_6_ in the IL-6 equation, and *k*_10_ *_M_* in the IL-10 equation were determined to be the most sensitive and therefore suitable for use in fitting the conditions of the experimental data.

To assess the performance of the model, a linear least squares (LLS) regression on each cytokine was utilized to compare the simulated results to the LTA- and Pep-G based immune response studies. The results of the regressions and corresponding regression validation parameters are given in **Table 3**. An *F*-test was performed on each regression to test the fit of the linear regression model. Each *F*-test resulted in a *p*-value significant to the ⍺ = 0.05 level for all comparisons of simulated and experimental data for cytokine-inducer interactions except for the TNF-⍺ and LTA interaction (significant to ⍺ = 0.10 level). This inconsistency was attributed to an outlier in the experimental TNF-⍺ LTA data, leading to a regression with a lower coefficient of determination. Based on the output of the statistical model, the cellular and cytokine mathematical model formulated under septic conditions accurately predicts whole human blood *ex vivo* conditions. Further development and clinical trial data will allow for additional cross-validated statistical algorithms to be run by splitting subjects into training, validation, and test sets for regressive modeling.

The linear regression models are given with 95% confidence intervals with a slope of 1 indicative of a perfect fit between the simulated concentrations and experimental results. Within one standard deviation, all of the cytokines fit the model except for IL-6 induced by LTA. The root mean squared error (RMSE), coefficient of determination, R^2^, and the adjusted coefficient of determination were also calculated to estimate the error distribution and variability of each regression (**Table 3**). The results of the statistical analysis indicate significant model similarities to the *ex vivo* experimental results and thus validate the accuracy of the simulation through clinical data.

**Figure 8:**
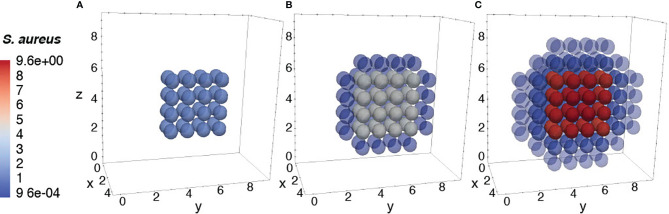
Diffusion of the bacteria at different time periods. Initial condition of bacteria injected only at the center of the domain **(A)**, after 12h of simulation **(B)**, and after 24h of simulation **(C)**.

**Figure 9:**
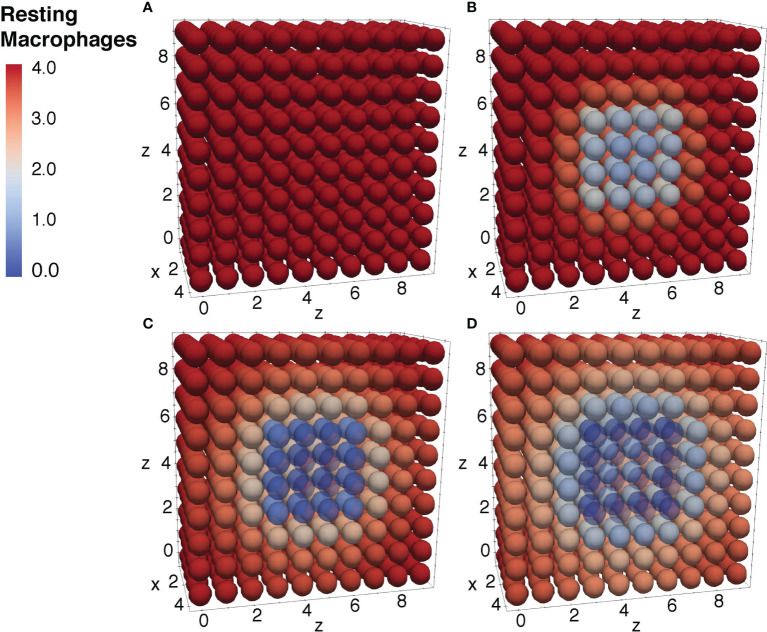
Diffusion of the resting macrophages at different time periods. Initial condition of the resting macrophages **(A)**, after 3h of simulation where the values decrease as a result of change of state from resting to activated **(B)**, after 12h of simulation **(C)**, and after 24h of simulation **(D)**.

**Figure 10:**
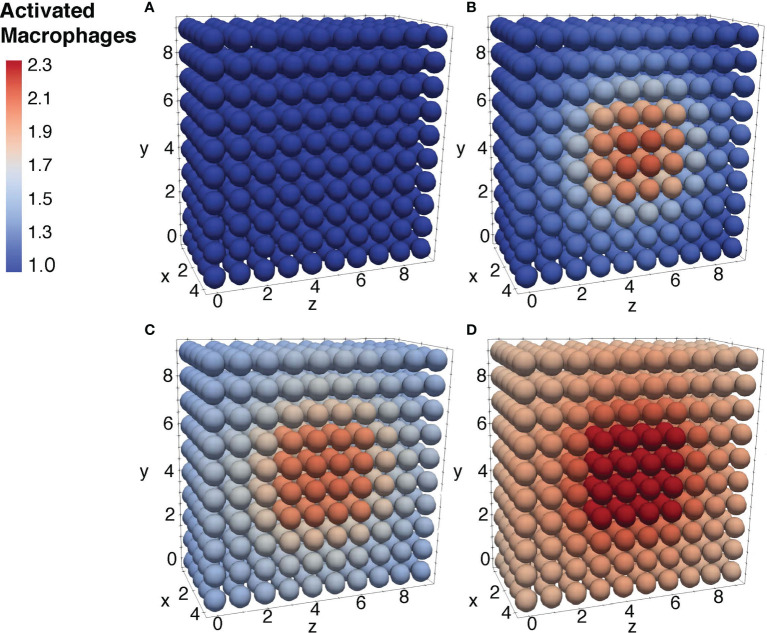
Diffusion of the activated macrophages at different time periods. Initial condition of the resting macrophages **(A)**, after 3h of simulation where the values increase as a result of change of state from resting to activated **(B)**, after 12h of simulation **(C)**, and after 24h of simulation **(D)**.

To further ensure the authenticity of the simulation in a biological setting, the spatial domains and diffusion within the 24-hour period were measured from the initial injection of the antigen in a small portion of tissue. The overall change in the levels of bacteria, resting macrophages, and activated macrophages are shown in **Figures 8**–**10**. This discretized domain of the 3-D diffusion model is a hexahedron representing 10 mm^3^ of tissue. This initial injection of antigen was represented in the center of the hexahedral domain of simulation (between 3 mm and 7 mm over the axes). Initially, the presence of macrophages is equally distributed over the tissue. Following their initial interaction with *S. aureus* or its cell wall components, the resting macrophages are activated, resulting in the production of cytokines and chemokines. Acting as antigen-presenting cells, activated macrophages travel to the nearest lymph node and present the antigen to lymphocytes, which initiate the activation and differentiation of T-lymphocytes and B-lymphocytes into T-helper 2 lymphocytes and plasma cells, respectively. All images show a cut view of the volume along the x-axis in order to better visualize both the initial condition and the concentration and diffusion of bacteria and macrophages (**Figures 8**–**10**).

## 4 Discussion

In this study, we explored the cellular-cytokine relationships of TNF-⍺, IL-6, IL-8, and IL-10 in response to *S. aureus* by utilizing mathematical modeling to predict cytokine levels *in silico*, and clinical literature and statistical analysis to validate the results of the simulation.

*Cellular-cytokine interaction.* The cellular model by Quintela et al. outlines the relationships between *S. aureus* (*A*), activated macrophages (*M_A_*), resting macrophages (*M_R_*), and antibodies (*F*) while the cytokine model by Brady et al. combines activated and resting macrophages with TNF-⍺, IL-6, IL-8, and IL-10 (Quintela et al., 2014; Brady et al., 2016). This model seeks to combine these models by the association of activated macrophages.

*Sensitivity analysis and parameter estimation.* This simulation validates the clinical experimentation from Wang et al. and Yao et al. (Yao et al., 1996; Wang et al., 2000). To scale the results of the clinical results, the parameters for each ODE was analyzed and modified using a min search nonlinear optimization to yield meaningful comparison.

*Model assumptions and limitations.* The limitations on the accuracy and precision of the model are discussed. This simulation does not account for factors such as neutrophil flux, complement response, humoral immune response effects, or variation between individuals, among other factors too complex to mathematically model and validate using clinical data.

*Future studies.* While this model seeks to expand the depth of current immune system models, the model remains incomplete due to the limitations in mathematical immune system research. The future expansions of the model open new pathways for new immune system research and may facilitate large-scale *in silico* pharmaceutical testing.

### 4.1 Cellular-Cytokine Interaction

This model combines the cellular model by Quintela et al. and the cytokine model by Brady et al. (Quintela et al., 2014; Brady et al., 2016). The cell model predicts the relationships between *S. aureus* (*A*), activated macrophages (*M_A_*), resting macrophages (*M_R_*), and antibodies (*F*), while the cytokine model studies how changes in activated and resting macrophages impact cytokine dynamics for TNF-⍺, IL-6, IL-8, and IL-10 (Quintela et al., 2014; Brady et al., 2016).

To reconcile the difference between the cell model, predicted as a function of time and space, and the cytokine model, which only varies with time, the activated macrophages were integrated into average activated macrophages and implemented into the cytokine model. In order to ensure both models operate within the same time frame, the concentrations of the initial bacteria and activated macrophages reported by Brady et al. (Brady et al., 2016) were scaled to match those reported in the *ex vivo* studies by Wang et al. and Yao et al. (Yao et al., 1996; Wang et al., 2000). Results of this parameter scaling demonstrated that we were able to match the model to all data metrics with statistical significance (*p*values <0.05) (**Table 3**) (Yao et al., 1996; Wang et al., 2000).

For future studies, a similar approach can be used to adapt the model to other experimental settings, e.g. to study the effect of varying macrophage destruction, flux, and phagocytosis within specific individuals (Emam et al., 2019). While the current simulation does not have the ability to accurately predict individual cytokine response, it has the capacity to model an average response to *S. aureus*, and it holds the potential to have a more profound impact with continued expansion in future studies.

### 4.2 Sensitivity Analysis and Parameter Estimation

Sensitivity analysis is an interpretable and adaptable tool used to provide insight into computational immunology studies investigating different components of the immune system to understand the extent of the spatial-temporal variables and parameters at play. This procedure has been shown to provide insight in computational immunology studies investigating different components of the immune system and their activation in response to a pathogen (Chen et al., 2019; Faro et al., 2019).

This coupled simulation is the product of sensitivity analysis using parameter estimation and model fitting to *ex vivo* data from Wang et al. and Yao et al. (Yao et al., 1996; Wang et al., 2000). Results of this analysis indicated that *k*_8_ *_m_*, which describes the upregulation of IL-8 by the activated macrophages, as the most influential parameter and was subsequently modified to mirror the clinical data. Throughout the analysis, the initial concentrations of *S. aureus* and the cytokines were scaled to yield meaningful comparison.

### 4.3 Model Assumptions and Limitations

While a comprehensive model would be powerful on a global scale, the current simulation is limited by the lack of clinically backed mathematical models of the human immune system.

The reconciliation of utilizing *S. aureus* (a gram-positive bacteria), from the cell model, and LPS (a component in gram-negative bacteria), used in the cytokine model, are discussed previously. The body’s response to the bacteria may change based on the damage inflicted by the pathogen including sepsis and septic shock. From a mathematical and clinical property standpoint, there are minimal differences in the activation of the immune system cells and cytokine expression between *S. aureus* and LPS, allowing for a simplified mathematical model coupling (Feezor et al., 2003; Fournier and Philpott, 2005). However, future studies investigating each PAMP independently would aid in further confirming or validating our findings, and additionally may need to incorporate the complementary effects of LTA and PepG in *S. aureus* and LPS in gram-negative bacteria to model the array of pro-inflammatory (TNF-α and IL-6), anti-inflammatory (IL-10), and neutrophil chemoattractant (IL-8) responses under septic conditions (De Kimpe et al., 1995; Fournier and Philpott, 2005).

While the simulation results accurately predict *ex vivo* data, it should be noted that several assumptions were needed throughout the design of the model. The concentrations of *S. aureus*, LTA, and PepG are assumed to be proportional at low doses based on their activation of cytokines in the human immune response. Furthermore, toll-like receptors (TLRs) found in the membrane and cytosol of macrophages are the primary sentinels of PAMPs, and we assume a proportional relationship between macrophage concentration and PAMP recognition. Our model considers circulatory immune elements and does not consider intercellular or genetic regulatory aspects of immune response. (De Kimpe et al., 1995; Fournier and Philpott, 2005; Pigozzo et al., 2013).

Although the self-regulatory cytokine network model can respond to higher concentrations of *S. aureus (6)*, this simulation utilized low concentrations of *S. aureus* similar to those found in *ex vivo* experimental conditions. Higher concentrations of *S. aureus* cause rapid increases in cytokine and cellular responses due to tissue damage corresponding to the endotoxicity of gram-positive bacteria. However, these situations were omitted due to the low concentrations of *S. aureus* simulated in this work and insufficient data to validate increased concentrations.

The neutrophil flux into the tissue was also omitted from this simulation due to the lack of clinical experiments and presence of high variability between subjects (Spaan et al., 2013). *S. aureus*-neutrophil interactions are human-specific and may influence the way this model predicts average cytokine levels. Future simulations may incorporate the effects of neutrophils in the IL-8 ordinary differential equation as additional studies and *ex vivo* experiments focusing on their interactions emerge (Fournier and Philpott, 2005).

To simplify the complexity of the bacterial complement response, this model does not incorporate the relevant complement proteins. Complement response of the human body to pathogens such as *S. aureus* plays a role in the ability of the human body to activate chemoattractants for phagocytosis of the bacteria (Fournier and Philpott, 2005; Laarman et al., 2010). The complement response has variable cytokine effects on the human immune response based on the extent and type of inflammatory condition, which further complicates its role in cytokine production and mediation (Fournier and Philpott, 2005). Moreover, complement, cytokine, and chemokine responses have overlapping biological effects on the body under septic conditions, and are therefore omitted from this model (Charchaflieh et al., 2012).

The effect of *S. aureus* on the humoral immune response is another limitation of this study given the insufficient findings of how that mechanism can be modeled (Smith et al., 2011). The pathogen is known to suppress the humoral immune response by means of the protein Sbi and therefore can allow extended survival of the bacteria (Smith et al., 2011). The effects of the protein Sbi on the concentration of bacteria inputted in the system were omitted from this model to solidify the cellular-cytokine interactions within the cellular model.

Macrophage destruction and flux are used as constants as a generalization in this model. Although these terms are human-specific and vary in response to infection by *S. aureus*, this model simulates the average response. Moreover, many components of the human immune response vary across populations. This complex problem of variability presents a challenge to mathematical modeling and is not addressed in this model. Therefore, certain variabilities such as macrophage destruction and flux were generalized to an average value to simplify the complexity of a human model. Understanding the different sources and mechanisms of action allows simulations to be used as predictive models to limit the scope of assumptions. One possible solution to account for patient-specific variation may be through estimating parameters individually for each dataset, as discussed in detail in Brady et al. (2016).

While this simulation can predict the average cytokine response to *S. aureus*, it does not account for all of the individualized variabilities within each immune response. This model aims to simplify the cytokine dynamic and become a foundation for future immune system expansion with increased immune system research. The model remains confined by the limited number of relevant mathematical immune system models and the availability of experimental data for validating more complex models. Continuous *in vivo* and *ex vivo* research into the role of the human immune system in response to a pathogen will provide additional data for validation and expansion of this model.

### 4.4 Future Studies

The human immune system, similarly, to many other body systems, is adversely affected by the aging process. Studies have shown age-associated alterations in immune system mechanisms such as a decreased T cell activation, reduced neutrophil efficiency, and altered toll-like receptor 1 (TLR1) expression (Oh et al., 2019) limit the ability of the immune system to eliminate foreign pathogens by reducing surface expression and effectivity (Khoa et al., 2003). In particular, the body’s cytokine and chemokine levels change with age, leading to chronic inflammation and progression of other degenerative diseases (Ponnappan and Ponnappan, 2011). This improved model along with other mathematical models simulating immune system functions may facilitate a better understanding of inflammatory responses and mechanisms that lead to the degradation of the human immune system and may lead to effective solutions towards preserving the functions of the immune system during the aging process.

Mathematical and computer models of the complex relationships within the immune system can open larger avenues for pharmaceutical and biochemical research that can be used to combat immune system diseases (Wang and Deisboeck, 2014). By incorporating both ordinary and partial differential equations, this simulation is able to provide a more realistic representation of the complex relationships within the immune system and, with further expansion, may serve as a vehicle for drug testing *in silico*.

While not incorporated into this model, pharmaceutical drugs such as FOM (Michalopoulos et al., 2011), CAM, (PubMedHealth, 2018) and DEX (Johnson et al., 2021) may significantly alter the results of this simulation. FOM inhibits PepG production in the cell wall of gram-positive bacteria, such as *S. aureus*, while also enhancing the production of IL-6 and IL-10. This may alter the cytokine production rate in this model as decreased *S. aureus* production eliminates the need for other cytokines in the immune response. With FOM, IL-6 and IL-10 production may increase.

The antibiotic, CAM, down-regulates the production of TNF-⍺, IL-6, and IL-8, which would lead to a measurable decrease in cytokine production. This cytokine inhibitor can significantly affect the simulation results, causing downward shifts of multiple cytokine values. Lastly, DEX, a steroid that hinders the immune system in the presence of inflammation, reduces the production of both IL-6 and IL-10, resulting in reduced TNF-⍺, IL-6, IL-8, and IL-10 concentrations.

Future expansions of this model may provide a clearer image of the various interactions within the immune system, facilitate a better understanding of the mechanisms that lead to the degradation of the immune system during the aging process, and may become a vehicle for *in silico* clinical trials (Swain and Nikolich-Zugich, 2009). This model is foundational and future clinical research is encouraged to characterize additional cytokines and cell behavior to create a more comprehensive and accurate understanding of the immune system. In particular, IL-12 is a proinflammatory cytokine that forms a vital link between the innate and adaptive immune system and future work would benefit greatly from accurately modeling IL-12 behavior (Trinchieri, 2003).

## Data Availability Statement

The original contributions presented in the study are included in the article. Further inquiries can be directed to the corresponding author.

## Ethics Statement

The studies involving human participants were reviewed and approved by the Regional Committee on Health Research Ethics (protocol ID H-3-2012-011) and the Regional Data Monitoring board (ID j-2007-58-0015, local 30-0766), and reported to clinicaltrials.gov (NCT01592526). The patients/participants provided their written informed consent to participate in this study.

## Author Contributions

KT contributed to the design, analysis, drafts, format, simulation, and overall production of the paper. SG contributed to the design and conception of the study. BQ, RS, and ML contributed to the mathematical modeling and coding aspects of the study. MO contributed to the mathematical modeling, writing and proofreading of the paper. JC contributed to the design, analysis and drafts of the paper. JJ contributed to the research collection, review, and major editing of the paper. PB contributed to the research collection and references of the paper. ES contributed to the research collection of the paper. WP contributed to the statistical analysis and simulation of the paper. NM contributed to the equation review and overall model parameters. MM contributed to the sensitivity analysis and PDE diffusion model. All authors contributed to the article and approved the submitted version.

## Funding

This study was funded by the Center for Research and Education in Aging, UC Berkeley Endowment.

## Conflict of Interest

The authors declare that the research was conducted in the absence of any commercial or financial relationships that could be construed as a potential conflict of interest.

## Publisher’s Note

All claims expressed in this article are solely those of the authors and do not necessarily represent those of their affiliated organizations, or those of the publisher, the editors and the reviewers. Any product that may be evaluated in this article, or claim that may be made by its manufacturer, is not guaranteed or endorsed by the publisher.
